# Serological Screening for Coronavirus Infections in Cats

**DOI:** 10.3390/v11080743

**Published:** 2019-08-13

**Authors:** Shan Zhao, Wentao Li, Nancy Schuurman, Frank van Kuppeveld, Berend-Jan Bosch, Herman Egberink

**Affiliations:** Virology Division, Department of Infectious Diseases & Immunology, Faculty of Veterinary Medicine, Utrecht University, Yalelaan 1, 3584CL Utrecht, The Netherlands

**Keywords:** coronaviruses, cats, spike protein, ELISA, virus neutralization, cross-species transmission, cross-reaction

## Abstract

Coronaviruses (CoVs) are widespread among mammals and birds and known for their potential for cross-species transmission. In cats, infections with feline coronaviruses (FCoVs) are common. Several non-feline coronaviruses have been reported to infect feline cells as well as cats after experimental infection, supported by their ability to engage the feline receptor ortholog for cell entry. However, whether cats might become naturally infected with CoVs of other species is unknown. We analyzed coronavirus infections in cats by serological monitoring. In total 137 cat serum samples and 25 FCoV type 1 or type 2-specific antisera were screened for the presence of antibodies against the S1 receptor binding subunit of the CoV spike protein, which is immunogenic and possesses low amino acid sequence identity among coronavirus species. Seventy-eight sera were positive for antibodies that recognized one or more coronavirus S1s whereas 1 serum exclusively reacted with human coronavirus 229E (HCoV-229E) and two sera exclusively reacted with porcine delta coronavirus (PDCoV). We observed antigenic cross-reactivity between S1s of type 1 and type 2 FCoVs, and between FCoV type 1 and porcine epidemic diarrhea virus (PEDV). Domain mapping of antibody epitopes indicated the presence of conserved epitope(s) particularly in the CD domains of S1. The cross-reactivity of FCoV type 1 and PEDV was also observed at the level of virus neutralization. To conclude, we provide the first evidence of antigenic cross-reactivity among S1 proteins of coronaviruses, which should be considered in the development of serological diagnoses. In addition, the potential role of cats in cross-species transmission of coronaviruses cannot be excluded.

## 1. Introduction

Coronaviruses (CoVs) are enveloped viruses with a positive-stranded RNA genome and classified into four genera (*alpha-, beta-, gamma- and deltacoronavirus*) within the subfamily *Orthocoronavirinae* in the family *Coronaviridae* of the order *Nidovirales*. CoVs are found in a variety of mammals and birds, in which they can cause respiratory, enteric and systemic infections [1,2,3]. Additionally, CoVs have proven ability for cross-species transmission, exemplified by the emergence of severe acute respiratory syndrome (SARS) coronavirus in 2002/2003, and of the Middle-East respiratory syndrome (MERS) coronavirus in 2012 [4]. Both viruses belong to the *Betacoronavirus* genus and have an animal origin. SARS coronavirus crossed over from bats via intermediate hosts to humans, became human-adapted and quickly spread worldwide before its containment. MERS coronavirus recurrently enters the human population via its dromedary camel reservoir host, with limited, non-sustained human-to-human transmission particularly in healthcare settings [5,6,7]. Apart from SARS- and MERS-CoV, all four globally endemic human CoVs (HCoV-OC43, HCoV-NL63, HCoV-229E and HCoV-HKU1) originate from animals [8,9,10,11]. In addition, cross-species transmission potential of CoVs is also illustrated by the occurrence of chimeric coronaviruses that resulted from recombination events between feline CoVs (FCoV) and canine CoVs (CCoV) [12,13].

In order to get insight into the frequency of interspecies transmission of coronaviruses within and between animal and human populations and the risk of subsequent development of a pandemic, it is useful to screen for coronavirus infections in animal species; especially those that are in close contact with humans. Serological assays that can detect virus-specific antibody responses against infection play an important role in these epidemiological studies [14].

Cats live in close contact with humans and often roam around freely in the environment. Hence cats are an interesting species to study for infections with coronaviruses. Infections with feline coronaviruses (FCoVs) are recognized and widespread [15,16]. FCoVs are classified into two types, type 1 and type 2, based on the genetic and antigenic difference of their spike (S) protein [17]. In the field, the majority of FCoV infections are caused by FCoV type 1, while FCoV type 2, derived from recombination events of type 1 FCoVs and CCoVs obtaining the S gene and some flanking regions of CCoVs, is less prevalent [18,19]. Depending on the virulence of the FCoV strain and the immune response of the cat, the clinical presentation can range from apparently asymptomatic, through diarrhea, to full-blown feline infectious peritonitis [20]. FCoVs are members of the genus *alphacoronavirus*, to which also HCoV-229E, porcine transmissible gastroenteritis virus (TGEV), and CCoV belong. The latter three viruses and FCoV type 2 have been proven to use feline aminopeptidase N (fAPN) as a functional receptor in vitro [21]. The receptor for type 1 FCoV has still not been identified [22]. Notably, previous studies have shown that HCoV-229E and CCoV could infect cats after experimental inoculation, causing an asymptomatic infection [23,24]. Thus, cats might potentially become naturally infected with CoVs of other species which may lead to virus-host adaptation e.g., mutation or recombination, resulting in emergence of novel coronaviruses and potentially new diseases [19,25]. The extent to which infections with CoVs of other species occur in the field, has not been explored in previous epidemiological studies of CoV infections in cats [15,26,27,28].

Being the main envelope protein of coronaviruses, the spike (S) protein mediates cell attachment and membrane fusion to allow viral entry. S functions as the main determinant of cell-, organ- and host-tropism. Additionally, it is also the major target of neutralizing antibodies. Spike comprises two functionally interdependent subunits, S1 and S2, with S1 responsible for receptor binding and S2 for membrane fusion [3,29]. The S1 subunit is the least conserved and the most variable immunogenic antigen between coronavirus species [30]. Therefore, the S1 subunit is well suited as an antigen to screen for coronavirus type specific antibodies [31].

In this study, CoVs infection in cats were detected through profiling antibody presence in serum samples from cats. Recombinant CoV spike S1 subunits of different animal and human CoVs were expressed in a mammalian expression system and used for screening of cat sera for the presence of antibodies against the respective proteins. Positive samples were also tested by virus neutralization assays to support the specificity of the reaction [32,33,34]. This investigation intends to extend our knowledge of CoV epidemiology, potential reservoirs, and cross-species transmission.

## 2. Materials and Methods

### 2.1. Serum Samples

Specific FCoV type 1 and FCoV type 2 sera were obtained from specific pathogen free (SPF) cats previously infected with strain UU2 or RM and FIPV-1146 respectively [35,36]. In addition, for the serological survey, 137 feline sera were retrieved from the serum bank in our lab. These had all been collected from cats in the Netherlands. Most of the samples (>80%) were from a study on antibody titer testing for feline panleukopenia virus. The other samples were send to our lab for FIP or FeLV-FIV diagnostics. Sera of uninfected SPF cats were included as negative controls. All samples were stored at −20 °C until analysis.

### 2.2. Cells and Viruses

African green monkey kidney cells (Vero-CCL81), human hepatoma cells (Huh7), pig kidney epithelial cells (LLC-PK1), human embryonic kidney 293 cells stably expressing the SV40 large T antigen (HEK-293T) were maintained in Dulbecco modified Eagle medium (DMEM, Lonza, Basel, Switzerland) supplemented with 10% fetal bovine serum (FBS, Bodinco, Alkmaar, The Netherlands). 

Virus strains used in this study have been described previously [37,38,39]. Briefly, recombinant porcine epidemic diarrhea virus (PEDV) (rPEDV-S^DR13^-GFP) was propagated and titrated in Vero cells, and HCoV-229E in Huh7 cells. PDCoV was propagated and titrated in LLC-PK1 cells, but supplemented with 1 μg/mL TPCK-treated trypsin (Sigma-Aldrich, Inc., St Louis, MO, USA) in DMEM.

### 2.3. Plasmids Constructs and Recombinant Protein Expression

Synthetic sequences of 12 coronavirus spike S1 subunits (HCoV-HKU1 (GB: YP_173238.1), MERS-CoV (GB:YP_009047204.1), SARS-CoV (GB: AAX16192.1), HCoV-OC43 (GB: AAR01015.1), HCoV-229E (GB: NP_073551.1), HCoV-NL63 (GB: YP_003767.1), TGEV (GB: ABG89325.1), PEDV (GB: AOG30832.1), BCoV (GB: P15777.1), PDCoV (GB: AML40825.1), FCoV type 1 (GB: FJ938060.1), FCoV type 2 (GB: AY994055.1)) and different domains of PEDV S1 subunit (S1^0^ and S1^A-D^, as identified and described in [40]) were cloned into pCAGGS expression plasmids as described previously [41]. Similarly, the expression constructs encoding chimeric proteins in which S1s were fused to the Fc domain of mouse IgG2a. For protein production, HEK-293T cells were transfected with plasmid DNA conjugated to polyethyleneimine (Polysciences, Inc., Warrington, PA, USA). At 8–16 h post transfection, inoculum was removed and the transfection mixture was replaced by 293 SFM II expression medium (Gibco®, Life Technologies Inc., Grand Island, NY, USA). At 6–7 days post transfection, cell supernatants were harvested and proteins were collected by Protein A Sepharose beads (GE Healthcare Bio-Sciences AB, Uppsala, Sweden). Proteins were then eluted with 0.1 M citric acid, pH 3.0 and neutralized with 1 M Tris-HCl, pH 8.8. Concentrations of proteins were assessed by Nanodrop spectrophotometry (ThermoFisher Scientific Inc., Waltham, MA, USA) and confirmed by sodium dodecyl sulphate polyacrylamide gel electrophoresis (SDS-PAGE) with bovine serum albumin (BSA, BioIVT, West Sussex, UK) as standard. Typical yields for proteins were 0.2–0.5 mg/mL. For long term storage, proteins were stored at −80 °C upon usage.

### 2.4. FCoV S Structure Modelling and S1 Domain Expression

To study the potential cross-reaction between FCoV type 1 and 2 in more detail, models of FCoV type 1 (strain: UU2; GenBank accession no.: FJ938060.1) and FCoV type 2 (strain: 79-1146; GenBank accession no.: AY994055.1) S proteins were generated via the automated protein structure SWISS-MODEL Homology Modelling server (https://swissmodel.expasy.org/) [42] using the elucidated HCoV-NL63 Cryo-EM structure (PDB code: 5SZS) as the input model. Figures were made with PymoL (The PyMOL Molecular Graphics System, Version 1.0 Schrödinger, LLC.). FCoV S1 domains of both type 1 and 2, namely S1^0-CD^, were expressed as murine Fc fusion proteins in HEK-293T cells as described above.

### 2.5. Enzyme-Linked Immunosorbent Assay (ELISA)

High binding microtiter plates (Greiner Bio-one BV, Alphen aan den Rijn, The Netherlands) were coated overnight at 4 °C with equal molar amount of protein (0.25 pmol per well, diluted in phosphate buffered saline (PBS, pH 7.4)). After three washes with washing buffer (PBS containing 0.05% Tween-20), the plates were blocked for 2 h at 37 °C with blocking buffer (PBS containing 5% milk powder (Protifar, Nutricia, Zoetermeer, The Netherlands), 0.05% Tween-20). Protein coating efficiency was assessed by binding of anti-mouse IgG antibodies in a direct ELISA, and confirmed the equimolar coatings of all proteins. To detect antigenic reaction with serum samples, sera were tested in duplicate at a 1:200 dilution in blocking buffer, and then incubated in the plates at 37 °C for 1 h. After washing, plates were incubated with a 1:4000 diluted horseradish peroxidase (HRP)-conjugated goat anti cat IgG (Rockland Immunochemicals, Inc., Pottstown, PA, USA) at 37 °C for 1 h. The peroxidase reaction was then visualized via adding TMB Super Slow One Component HRP Microwell Substrate (BioFX®, Surmodics IVD, Inc., Eden Prairie, MN, USA) for 10 min. Reaction was stopped with 12.5% sulfuric acid and optical densities (OD) were measured at 450 nm. Negative sera (from uninfected SPF cats) were included to determine the ELISA cut-off values; sera with OD values higher than 5-fold the OD of negative sera were considered positive. All 12 S1 proteins were coated on the same ELISA plates making it easy to screen and compare the OD values of individual sera in one assay. Hereby we excluded the sera that give high background OD values against all proteins being considered false positive.

### 2.6. Virus Neutralization Assay

Neutralization assays were performed with some of the CoVs to support the specificity of ELISA results. Cat sera were serially diluted 2-fold in DMEM and mixed 1:1 with rPEDV-S^DR13^-GFP, HCoV-229E or PDCoV (2000 50% tissue culture infective doses [TCID_50_]/mL). These mixtures were then incubated at 37 °C for 1h, and 100 µL of each mixture was used for inoculation with Vero, Huh7 and LLC-PK1 cell monolayers in 96-well plates, respectively. For PDCoV infection, TPCK-treated trypsin (Sigma-Aldrich, Inc., St Louis, MO, USA) was supplied to LLC-PK1 tissue culture medium at a final concentration of 1 μg/mL. At 2–5 days post infection, cytopathic effect (CPE) could be observed via microscopy. Virus neutralization titers (VNT) were expressed as the highest serum dilution resulting in 90% reduction of cytopathic effect (HCoV-229E and PDCoV) or virus-induced fluorescent cells (PEDV). Before virus neutralization, sera were inactivated through incubation at 56 °C for 30 min. Experiments were performed in triplicate. 

## 3. Results

### 3.1. CoVs Seroprevalence in Cats

**Figure 1:**
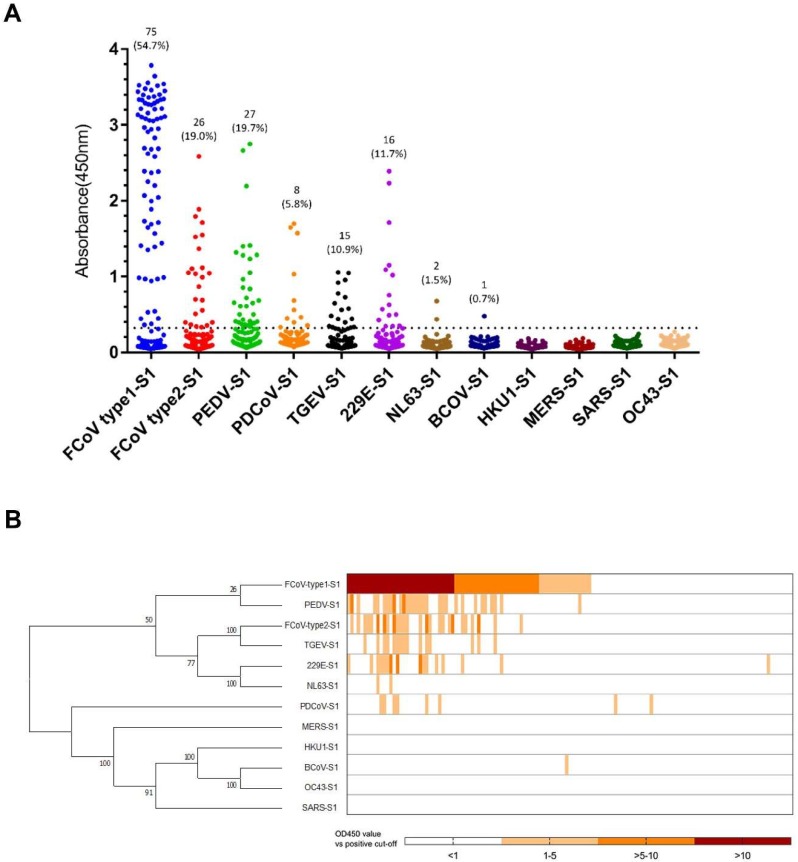
ELISA-reactivity of feline serum samples against S1 proteins of different coronaviruses. (**A**) Reactivity of feline serum samples (*n* = 137) against 12 coronavirus S1 antigens was determined by ELISA. The number and percentage of seropositive samples are indicated for each S1 protein. The dashed line is the positive cut-off. Each dot represents one individual sample within each antigen panel. (**B**) Relative ELISA (OD450 values divided by cut-off value) results are displayed as a heat map. Different S1 antigens were grouped by amino acid sequence phylogeny (left panel) using MEGA7. Each column of the heat map represents an individual sample, and columns were arranged in a descending order based on ELISA-reactivity against feline coronavirus (FCoV) type 1.

Feline sera (*n* = 137) were screened by indirect ELISA for antibody reactivity against 12 CoV S1 antigens. The OD values against these 12 antigens are shown in Figure 1. In total, 78 of the 137 sera (56.9%) contained anti-CoV antibodies, while 43 sera showed reactivity against more than one CoV S1 antigen. None of the samples had to be discarded because of reactivity against all of the proteins indicating a potential false positive result. The frequency of different combinations of CoV-S1 reactive samples is summarized in Table 1. Reactivity against eight out of 12 CoV S1 antigens could be observed, whereas none of the sera recognized the S1 protein of HCoV-HKU1, MERS-CoV, SARS-CoV and HCoV-OC43.

As expected, many sera were positive for FCoV S1, with 75 sera (54.7%) positive for FCoV type 1 and 26 sera (19.0%) for FCoV type 2 S1 (Figure 1). All of the FCoV type 2 S1 positive sera also tested positive for FCoV type 1 S1, while 15 of 26 FCoV type 2 S1 positive sera also reacted with TGEV S1. The FCoV type 2 S1 and TGEV S1 ELISA reactivities showed a strong nonparametric Spearman correlation (Spearman r = 0.84, *p* < 0.0001). With respect to this, we suggest that the TGEV S1 positivity was due to cross-reactivity of FCoV type 2 S1, as FCoV type 2 shows close antigenic and genetic relationship with TGEV (S1 shares 70.4% amino acid sequence identity). The remaining 11 FCoV type 2 S1 positive but TGEV S1 negative sera do react with FCoV type 1 S1. An explanation might be the cross-reactivity between FCoV type 1 and type 2. Remarkably, 40 feline sera were reactive with S1 proteins from human, porcine and bovine CoVs (Table 1), including HCoV-229E (16/137), HCoV-NL63 (2/137), PEDV (27/137), PDCoV (8/137) and BCoV (1/137). OD values of feline sera positive for HCoV-NL63 S1 and BCoV S1 were relatively low (Figure 1A). ELISA reactivity towards non-feline CoV S1 proteins might be explained by infection with the respective or related CoVs or by the presence of cross-reacting antibodies, although there was low sequence identity (< 32.8%) between S1 proteins of FCoV type 1 and related non-feline coronaviruses (for the complete comparison of S1 sequence identities, see Table 2). Yet, all of PEDV-S1 positive sera were also positive for FCoV type 1 S1 (Figure 1B, Table 1). The ELISA results of FCoV type 1 S1 and PEDV S1 showed a strong nonparametric Spearman correlation (Spearman r = 0.83, *p* < 0.0001). Thus, this might indicate the occurrence of antibody cross-reactivity against FCoV type 1 and PEDV S1 antigens. Many of the HCoV-229E and PDCoV S1 positive sera also reacted with FCoV type 1 S1, but no strong nonparametric Spearman correlation was observed (HCoV-229E, r = 0.216; PDCoV, r = 0.307). One feline serum only reacted with HCoV-229E S1, and two feline sera only recognized PDCoV S1. (Figure 1B, Table 1). This observation led us to hypothesize that cross-reactivity may not play a role in ELISA reactivity of these three sera, but that the three cats had been infected with these viruses or related viruses. 

### 3.2. Assessment of Cross-Reactivity for CoV S1s

In our screening, 43 samples were shown to be positive for two or more S1 proteins including FCoV type 1. The data prompted us to test different hypotheses which may explain this phenomenon: specific reaction through natural virus infection or reaction due to cross-reactivity with FCoV-S1 antigens. To explore this further, we employed 25 FCoV type 1 specific sera derived from specific pathogen free (SPF) cats that had been experimentally infected with FCoV type I strain RM (*n* = 9) or strain UU2 (*n* = 16). These sera were tested for their ELISA reactivity against seven CoV S1 proteins (excluding TGEV S1) that showed positive reactivity in the previous serological screening. As expected, all 25 sera were positive for FCoV type 1 S1 in our ELISA; interestingly, four samples also reacted with FCoV type 2 S1, and five samples with PEDV S1. No positive ELISA-reactivity was detected with S1 of HCoV-229E, PDCoV, HCoV-NL63 or BCoV (Table S1). Thus, FCoV type 1 infection could lead to the generation of antibodies that cross-react in the S1-ELISA with FCoV type 2 and PEDV S1 proteins. 

### 3.3. S1 Domain Mapping of Conserved Epitopes Shared between Type 1 and Type 2 Feline Coronaviruses 

**Figure 2:**
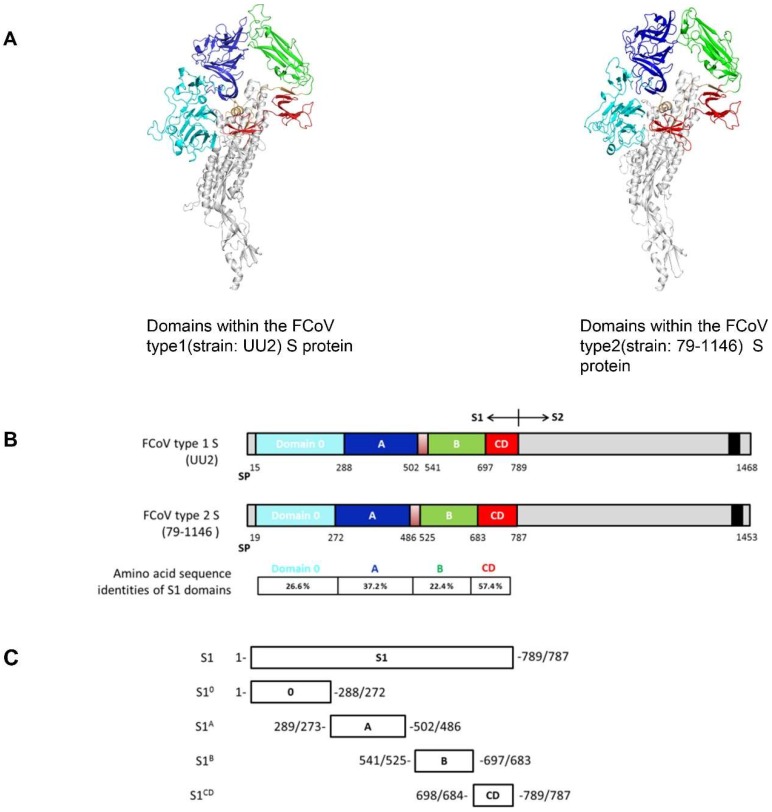
Domains within the FCoV type 1 and type 2 spike (S) proteins. (**A**) Structure model of two types of FCoV S trimer based on the HCoV-NL63 S structure were generated via the SWISS-MODEL Homology Modeling server using ProMod3. Figures were produced by PyMOL. Different domains of the S1 subunit of one protomer are colored, with S1^0^ shown in cyan, S1^A^ in blue, S1^B^ in green, and the domains S1^CD^ in red. The S2 part of the protomer is marked in light gray. (**B**) Schematic presentation of the FCoV type 1 (strain UU2) and type 2 (stain 79-1146) S protein with the signal peptide (SP), the S1 subunit (the domains are colored as described in the legend of Figure 2A) and the S2 subunit (the C-terminal transmembrane domain is indicated by a black box). Amino acid sequence identities between FCoV type 1 and type 2 S1 domains are indicated. (**C**) Diagram of the different S1 subdomains sequence. All S1 subdomains were C-terminally tagged with the Fc part of mouse IgG2a (not shown in the figure) and expressed as Fc fusion proteins.

**Figure 3:**
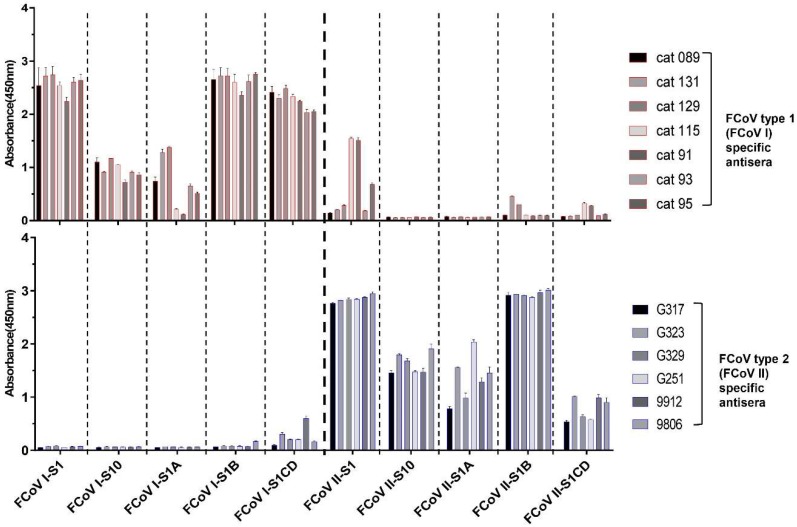
ELISA-reactivity of FCoV specific antisera against different S1 subdomains of FCoV type 1 and 2. Equimolar amount of purified S1 proteins and the four S1 subdomains were coated onto 96-well plates and antibody binding was determined by ELISA. The FCoV type 1 and 2 specific antisera used in the screening were derived from experimentally infected specific pathogen free (SPF) cats and are indicated at the right side of each panel, absorbance values and antigens in use are shown on the y- and x-axis, respectively. Graphs represent the mean values from three independently performed experiments. Standard deviations are indicated as error bars.

The ELISA cross-reactivity of FCoV type 1 specific sera with FCoV type 2 S1 antigens prompted us to map the domains responsible for cross-reaction within the S1 subunit. Hence, to identify domain borders within S1, we built homology-based models of both FCoV type 1 and type 2 spike using the related elucidated HCoV-NL63 cryo-EM structure as the template model. As shown in Figure 2A, continuous structural domains can be identified for the S1 subunit of both spikes, namely S1^0^, and S1^A^ through S1^D^. Amino acid sequence identities of these domains between FCoV type 1 and type 2 differ, ranging from 22.4% to 57.4% (Figure 2B). Several S1 proteins for both type 1 and type 2 FCoV-S1 comprising one or two domains were expressed and purified (Figure 2C). FCoV type 1 specific sera (*n* = 4 for strain RM and *n* = 3 for strain UU2) and type 2 (*n* = 6, strain 79-1146) specific sera were then tested against these proteins in ELISA format. The four FCoV type 1 specific sera that cross-reacted with S1 of FCoV type 2 again showed binding to FCoV type 2 S1. But the FCoV type 2 specific sera showed little to no reactivity against FCoV type 1 S1. As shown in Figure 3, the type specific antisera reacted with all of the homologous S1 domains, with S1 and S1^B^ of both type 1 and type 2 displaying the strongest reaction. Interestingly, the CD domain showed the highest level of cross-reactivity between FCoV type 1 and 2, in agreement with its highest sequence identity among S1 domains (Figure 3). The other three domains showed little to no cross-reactivity.

### 3.4. Assessment of Cross-Reactivity between FCoV Type 1 and PEDV 

**Figure 4:**
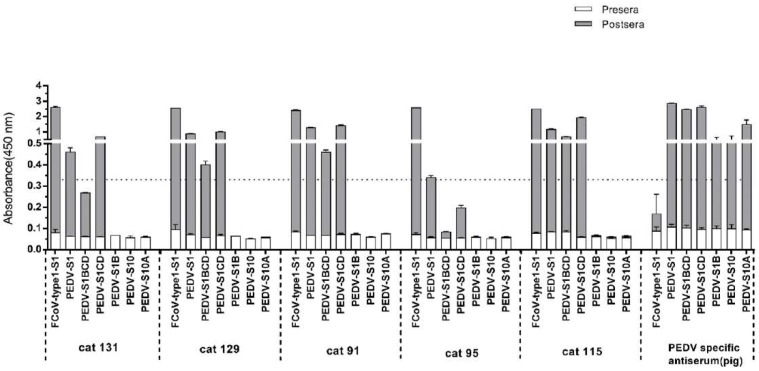
Reactivity of feline sera pre- and post FCoV type 1 infection to porcine epidemic diarrhea virus (PEDV) S1 domains determined by ELISA. Equal molar amount of purified S1 proteins and PEDV S1 subdomains were coated onto 96-well plates and antibody binding was tested by ELISA. Serum samples tested are indicated at the bottom of each panel. Moreover, absorbance at 450 nm and antigens are shown on the y- and x-axis, respectively. OD450 values of feline sera pre- and post FCoV infection were superimposed to one bar. Cat 91–131: sera from SPF cats pre- and post-experimental infection with FCoV type 1; PEDV specific antiserum: sera collected from a pig pre- and post-experimental inoculation with PEDV-S1. The dashed line shows the positive cut-off level. The graphs represent the means from three independent experiments. Error bars indicate standard deviations.

**Figure 5:**
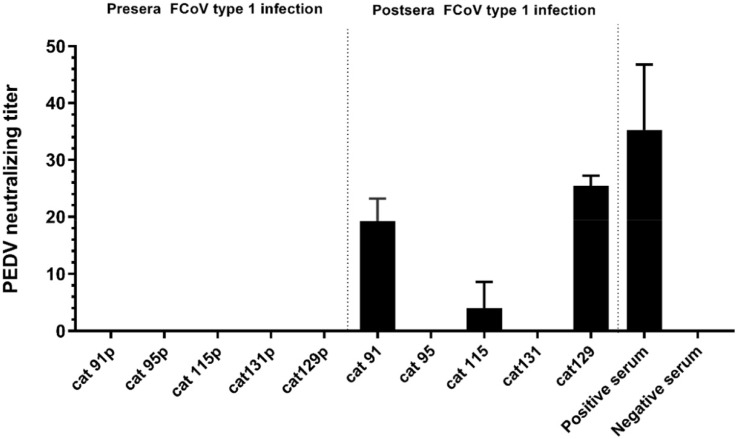
Neutralization of PEDV by cat antisera raised against FCoV type 1. rPEDV-S^DR13^-GFP was mixed 1:1 with serial dilutions of serum prior to inoculation of Vero cells. Three days post infection, cytopathic effect (CPE) could be observed and fluorescent cells were identified. The highest serum dilution inhibiting virus infections was recognized as the virus neutralization titer (VNT). The experiment was carried out in duplicate and repeated three times. Error bars indicate standard deviations. Sera were collected from SPF cats prior (cat 91–131p) and after (cat 91–131) experimentally inoculated with FCoV type 1. Positive serum: PEDV positive swine serum collected from the field; Negative serum: serum from FCoV negative SPF cat.

Because the FCoV type 1 specific cat sera also showed ELISA reactivity with PEDV-S1 (Table S1), we analyzed the reaction of the five PEDV-S1 positive cats in more detail. Samples were analyzed via ELISA using antigens comprising different PEDV-S1 domains, as described in our previous study [40]. Cat sera taken pre- and post- FCoV infection were collected and tested. As indicated in Figure 4, all five cats had developed PEDV-S1 reactivity to different extent after FCoV type 1 inoculation. Noticeably, all sera showed the highest OD values with the CD domain, while the other domains, including the S1^B^ containing the presumed receptor binding domain (RBD) [40], were non-reactive (Figure 4). On the other hand, the swine PEDV positive control serum exhibits strong reactivity against all PEDV-S1 domains. The next question we asked was whether FCoV type 1 specific sera could neutralize PEDV infection in tissue culture, as they showed no reactivity with the S1^B^ of PEDV spike. As shown in Figure 5, PEDV neutralizing antibodies were detected in three out of five FCoV type I specific cat sera. 

### 3.5. Evaluation of Virus Neutralization Capacity of HCoV-229E and PDCoV Positive Serum Samples

Several serum samples from field cats, but not virus-specific serum samples from FCoV inoculated SPF cats, were found to be ELISA positive for HCoV-229E (*n* = 16) and PDCoV S1 (*n* = 8) (Figure 1A). Also, a few feline sera displayed unique ELISA positivity for S1 of HCoV-229E (*n* = 1) or PDCoV (*n* = 2) (Figure 1B, Table 1). This could indicate that these antibodies were induced upon infection with these specific viruses. To corroborate the possibility of a natural infection in these cats with HCoV-229E or HCoV-229E-like viruses, we tested sera neutralization antibody titers. The results showed that one of the HCoV-229E S1 reactive feline sera was able to neutralize HCoV-229E infection (VNT = 32); no neutralization of PDCoV was detected for the all PDCoV-S1 positive sera. 

## 4. Discussion 

Coronavirus infections are endemic and ubiquitous in feline populations. Two viral types, type 1 and 2, are distinguished and both of them could well sustain themselves in the cat reservoir [15,43]. Both have been shown to have worldwide distribution, with the seropositivity rate up to 90% among animal shelter populations and in multi-cat households [20,44]. The majority of natural infections are caused by type 1 FCoVs, while in the field type 2 FCoVs are less common and mainly occur in Asia [15,28,45,46,47]. CoVs are generally considered to be host-specific; however, cross-species transmission does occur which may lead to incidental infections like the spillover of MERS-CoV from dromedary camel to humans, where humans function as an incidental and ultimately dead-end host [4]. But CoVs might also adapt to the new host exemplified by the animal origin of all four endemic human CoVs (HCoV-OC43, HCoV-NL63, HCoV-229E and HCoV-HKU1) [8,9,10,11]. Whereas in cats infections with FCoV are well recognized, studies regarding possible natural infections with other animal and human coronaviruses are lacking to the best of our knowledge. Knowing the genetic variability of coronaviruses and the use of orthologous receptors by non-feline CoVs, studies on cross-species transmission are desirable. This may provide insight regarding whether cross-species transmission does occur. In the present study we used the highly immunogenic S1 antigens to screen cat sera for the presence of antibodies against feline and non-feline coronaviruses, as a first indication of possible infections with these viruses. 

In our study, 78 of the 137 cat sera were shown to be seropositive for coronaviruses. The seropositive rate (54.7%) against S1 of FCoV type 1 is consistent with previous studies [15,47]. All of the FCoV type 2 S1 positive sera of naturally infected cats were also positive for FCoV type 1 S1, which might be the result of cross reaction between the two proteins, despite their low amino acid identity. ELISA with specific antisera from experimentally FCoV type 1 and type 2 infected cats showed that sera of several FCoV type 1 infected cats could cross-react with FCoV type 2 S1. Domain mapping ELISA results showed that FCoV type 1 specific sera react to different levels with the S1 domains of FCoV type 1 S1 protein, and also reacts with FCoV type 2 S1^CD^. Vice versa, FCoV type 2 specific sera also reacted with S1^CD^ of FCoV type 1. These observations pose a potential two-way cross-reactivity between S1^CD^ domains. Interestingly, in parallel with our findings on feline coronaviruses, we identified a number of samples that were seropositive against the S1 of PEDV, a viral pathogen that mainly replicates in the porcine intestinal epithelium. To study the possibility of cross-reaction, samples derived from pre- and post- FCoV infected cats were screened against PEDV S1 in ELISA. The reactivity found against PEDV S1 with FCoV specific sera shows that cross-reaction can occur at the level of domain S1^CD^; the other PEDV S1 domains showed no reaction with the FCoV positive sera. Judging from these observations, it seems that S1^CD^ plays an important role in cross-reaction between FCoV type 1 and 2, and also FCoV and PEDV. As S1^CD^ is the most conserved domain among FCoV and also between FCoV and other alphacoronaviruses (for a systematic assessment of sequence identities, see Table 3), it is reasonable to hypothesize that antibodies can develop against conserved epitopes within this region and subsequently cause cross-reaction. This should be taken into account when developing and interpreting serological assays.

Noticeably, ELISA reactivity among cat sera towards the N-terminal FCoV S1 domains 0 and A was less consistent and generally lower compared to whole S1, which seems to correlate with the higher antigenic variation in those domains found among FCoV type 1 strains [48] (Figure 3). Especially the sera from FCoV-RM infected cats (cat 91, 93, 95 and 115) showed lower OD values against S1^A^. This phenomenon could be explained as the samples displaying higher reactivity were from cats inoculated with FCoV-UU2 (cat 089, 131 and 129), the particular strain from which the S1 region was used as an antigen in the ELISA studies. In the meantime, the possibility of the variable ELISA reactivity might be due to the difference in individual antibody levels. In principle, the distinct antigenic reactivity of S1^0^ and S1^A^ between the two FCoV types might facilitate the development of a specific ELISA method which allows the serological discrimination of FCoV type 1 and type 2 infections in cats. 

In order to provide further insight regarding cross-reactivity between FCoV type 1 and PEDV, we performed virus neutralization assays. Cross-neutralization of PEDV infection could be observed for some of the feline FCoV type 1 post-infection sera, in contrast to the pre-infection serum counterparts. Since FCoV specific PEDV neutralizing sera did not react with PEDV S1^0^, S1^A^ or S1^B^, it is likely that the cross-neutralizing antibodies are targeting conserved epitopes in the S1^CD^ domain or the S2 subunit of the PEDV spike protein [49]. Given the unknown TGEV infection background of the PEDV positive pigs, the cross-reaction of PEDV specific sera against FCoV type 1 could not be explored in our study, as TGEV positive pig samples would certainly influence the outcome [12,18,50]. Of note, our findings cannot exclude the possibility that field cats might incidentally get naturally infected with PEDV or PEDV-like viruses, as there had been one report showing the detection of PEDV in one stray cat via PCR assay [51]. It would be interesting to include more sera of cats from pig farms in future studies.

Considering the fact that cats play an important role in human society and have constant interaction with humans, it is of interest to conduct serological surveys for possible reverse zoonosis of human pathogens. In our study S1 antigens of several human coronaviruses were included and this led us to identify HCoV-229E seropositive feline samples in our ELISA survey (Table 1); one serum in particular reacted solely with HCoV-229E S1 but not with any other coronavirus. Of the HCoV-229E S1 reactive feline sera one showed low neutralizing activity against HCoV-229E infection. This might suggest that positive cats were indeed exposed to HCoV-229E or related viruses. MERS seropositivity is also seen in other species besides the dromedary host [52]. Rare cases of seropositivity might be considered as spill-over infections from the dromedary camel reservoir. Similar (perhaps dead-end) spill-over infections of 229E from the human reservoir to cats might also occur. A similar principle could also apply for PDCoV, a porcine pathogen that emerged rather recently. Both HCoV-229E and PDCoV use APN as their receptor and have been reported to also be able to use feline APN for cellular entry [21,37]. Although reports are lacking regarding the natural infection of these two viruses in cats, HCoV-229E was shown to cause a priming effect of FCoV antibody in experimentally FCoV infected cats suggesting that infection occurred [24]. Therefore, the detection of antibodies against S1 of HCoV-229E in a portion of the cats might be specific and due to the exposure to HCoV-229E through daily interaction with humans. Eight cats were seropositive for PDCoV of which two cats were seropositive only for PDCoV and not for any other CoVs. This could be caused by infection with PDCoV or PDCoV-related viruses through avian sources, considering the fact that cats are natural avian predators and the presumed avian origin of PDCoV [2,37]. Our findings emphasize the potential role of cats as incidental hosts for non-feline coronaviruses and the need of in-depth study of naturally infected pathogens in cats. Besides serological studies, efforts should also focus on isolation and identification of these viruses in cats.

In conclusion, we presented a thorough serological survey in cats using S1 proteins of different animal and human coronaviruses. We demonstrated, despite the low amino acid identity, cross-reactivity between S1 proteins of FCoV type 1 and 2, and between that of FCoV type 1 and PEDV. This should be considered when developing FCoV serological assays as well as interpreting the results. Our observation that some feline sera displayed antibody reactivity exclusively against non-feline CoV S1 proteins warrant further research into the epidemiology and cross-species transmission of coronaviruses in cats and other animals that are in close contact with humans. Further large scale serological studies regarding coronaviruses infection across animal species using arrays of CoV S1 antigens can shed light into the hitherto unresolved host promiscuity of coronaviruses and the risk of cross-species transmission.

## Figures and Tables

**Table 1:** Numbers of positive cat samples and different combinations of reactivity found. The number of positive sera against each individual S1 is shown in the bottom row. Positive ELISA reactions are colored in orange. Cut-off value was determined as the 5-fold over the OD450 of negative sera.

S1 Antigens of Different Coronaviruses								Number of Cats (Total = 137)
FCoV Type1-S1	FCoV Type2-S1	PEDV-S1	PDCoV-S1	TGEV-S1	229E-S1	NL63-S1	BCoV-S1	
								59
								32
								6
								10
								1
								1
								2
								4
								2
								2
								1
								1
								1
								1
								1
								1
								1
								1
								1
								1
								4
								4
75	26	27	8	15	16	2	1	

**Table 2:** Percentage identity matrix of every pair of sequences of spike S1 subunit.

Sequence No.	Protein (Accession No.)	% Amino Acid Sequence Similarity with Protein of Sequence No.:										
		1	2	3	4	5	6	7	8	9	10	11
1	FCoV type1-S1 (FJ938060.1)											
2	FCoV type2-S1 (AY994055.1)	28.5										
3	PEDV-S1(AOG30832.1)	32.8	30.2									
4	PDCoV-S1 (AML40825.1)	23.1	26.7	26.5								
5	TGEV-S1 (ABG89325.1)	28.5	70.4	31.8	27.4							
6	HCoV-229E-S1 (NP_073551.1)	30.4	38.6	34.5	27.7	37.2						
7	HCoV-NL63-S1 (YP_003767.1)	28.4	30.4	29.6	26.7	31.7	51.9					
8	BCoV-S1 (P15777.1)	9.6	11.3	8.5	10.8	11.2	14	10.6				
9	HCoV-HKU1-S1 (YP_173238.1)	11	12.1	9.5	12.4	11.4	13.5	11.8	61.2			
10	MERS-S1 (YP_009047204.1)	9.2	9.2	7	6.6	9.4	7.9	8.1	15.6	17		
11	SARS-S1 (AAX16192.1)	6.5	8.9	9.1	8.6	8.9	11.4	8.5	17	17.9	15.5	
12	HCoV-OC43-S1 (AAR01015.1)	8.9	11.3	7.7	10.2	10.7	13.1	9.6	90.9	59.7	15.7	16.4

**Table 3:** Identities of amino acid sequences of FCoV type 1 (strain: UU2) S1 and S1 domains compared with the amino acid sequences of other alphacoronaviruses. (Identities are shown in %; NA: not available) The Genbank accession numbers of these viruses are as follows: FCoV type 1 (UU2), FJ938060.1; FCoV type 1 (RM), FJ938051.1; FCoV type 2, AY994055.1; TGEV, ABG89325.1; PEDV, AOG30832.1; HCoV-229E, NP_073551.1; HCoV-NL63, YP_003767.1.

	Amino Acid % Identity to FCoV Type 1(UU2)				
	S1	S1^0^	S1^A^	S1^B^	S1^CD^
FCoV type 1 (RM)	89.3	88.3	85	94.9	91.2
FCoV type 2	28.5	26.6	37.2	22.4	57.4
TGEV	28.5	24	38.2	23.1	57.3
PEDV	32.8	20.4	36.5	24.8	51.2
HCoV-229E	30.4	NA	36.5	21.4	35.6
HCoV-NL63	28.4	19.3	37.7	20.6	44.9

## Supplementary Materials

The following are available online at https://www.mdpi.com/1999-4915/11/8/743/s1, Table S1: ELISA reactivity (OD450 values) of 25 FCoV type 1 specific antisera against S1 antigens of different coronaviruses.
