# Supplementary material for: Serological Screening for Coronavirus Infections in Cats
Source: Viruses. 2019 Aug 13;11(8):743. doi: 10.3390/v11080743 (PMC6723642; doi:10.3390/v11080743)
Supplement: Supplementary file 1 [file viruses-11-00743-s001.pdf]

**Table S1.** ELISA reactivity (OD450 values) of 25 FCoV type 1 specific antisera against S1 antigens of different coronaviruses. Table represent the mean OD450 values from three independent experiments. Positive ELISA reactions are colored in orange. Cut-off value is 0.36 and was determine as the 5-fold over the OD of negative sera. The Genbank accession numbers of these viruses are as in table 3.

| serum samples | FCoV type 1 strains for infection | S1 antigens of different coronaviruses | | | | | | |
|---|---|---|---|---|---|---|---|---|
| | | FCoV type1 (UU2)-S1 | FCoV type2-S1 | PEDV-S1 | HCoV-229E-S1 | PDCoV-S1 | HCoV-NL63-S1 | BCoV-S1 |
| cat 91 | RM | 2.603 | 1.995 | 1.906 | 0.100 | 0.073 | 0.148 | 0.100 |
| cat 93 | RM | 2.923 | 0.231 | 0.198 | 0.116 | 0.075 | 0.096 | 0.120 |
| cat 95 | RM | 2.931 | 0.732 | 0.440 | 0.116 | 0.062 | 0.059 | 0.053 |
| cat 115 | RM | 2.619 | 1.786 | 1.817 | 0.128 | 0.116 | 0.084 | 0.114 |
| Cat 039 | RM | 1.221 | 0.173 | 0.153 | 0.053 | 0.098 | 0.099 | 0.093 |
| Cat 055 | RM | 1.220 | 0.129 | 0.077 | 0.085 | 0.091 | 0.114 | 0.065 |
| Cat 057 | RM | 1.001 | 0.173 | 0.099 | 0.066 | 0.072 | 0.093 | 0.092 |
| Cat 073 | RM | 1.905 | 0.240 | 0.123 | 0.096 | 0.109 | 0.113 | 0.064 |
| Cat 109 | RM | 1.650 | 0.189 | 0.116 | 0.068 | 0.060 | 0.082 | 0.092 |
| cat 129 | UU2 | 2.537 | 0.401 | 0.976 | 0.143 | 0.053 | 0.062 | 0.050 |
| cat131 | UU2 | 2.858 | 0.262 | 0.880 | 0.070 | 0.080 | 0.150 | 0.080 |
| cat 89 | UU2 | 2.246 | 0.125 | 0.244 | 0.071 | 0.106 | 0.087 | 0.130 |
| cat 024 | UU2 | 1.812 | 0.090 | 0.156 | 0.107 | 0.114 | 0.100 | 0.127 |
| cat 058 | UU2 | 1.607 | 0.110 | 0.139 | 0.074 | 0.069 | 0.071 | 0.115 |
| cat 066 | UU2 | 1.292 | 0.065 | 0.066 | 0.101 | 0.144 | 0.080 | 0.085 |
| cat 070 | UU2 | 1.924 | 0.102 | 0.090 | 0.091 | 0.086 | 0.122 | 0.050 |
| cat 164 | UU2 | 1.151 | 0.095 | 0.074 | 0.085 | 0.078 | 0.099 | 0.051 |
| cat 178 | UU2 | 1.371 | 0.127 | 0.147 | 0.114 | 0.082 | 0.081 | 0.068 |
| cat 188 | UU2 | 1.277 | 0.095 | 0.078 | 0.129 | 0.056 | 0.056 | 0.068 |
| cat 200 | UU2 | 0.640 | 0.096 | 0.073 | 0.142 | 0.126 | 0.096 | 0.060 |
| Cat 1 | UU2 | 2.091 | 0.263 | 0.144 | 0.120 | 0.149 | 0.091 | 0.077 |
| Cat 2 | UU2 | 1.846 | 0.172 | 0.155 | 0.090 | 0.083 | 0.100 | 0.144 |
| Cat 3 | UU2 | 1.566 | 0.117 | 0.082 | 0.058 | 0.074 | 0.051 | 0.075 |
| Cat 4 | UU2 | 2.284 | 0.099 | 0.157 | 0.054 | 0.094 | 0.134 | 0.063 |
| Cat 5 | UU2 | 2.319 | 0.105 | 0.099 | 0.095 | 0.067 | 0.116 | 0.058 |
